# Supplementary material for: Therapeutic Role of Polyphenol Extract from Prunus cerasifera Ehrhart on Non-Alcoholic Fatty Liver
Source: Plants (Basel). 2024 Jan 18;13(2):288. doi: 10.3390/plants13020288 (PMC10821496; doi:10.3390/plants13020288)
Supplement: Supplementary file 1 [file plants-13-00288-s001.zip › plants-2789055-supplementary.pdf]

Supplementary Materials and Methods:

S1. NAS semi-quantitative scoring system

Hepatocellular steatosis: 0 points (< 5%); 1 points (5% to 33%); 2 points (34% to 66%); 3 points (> 66%). Intralobular inflammation (necrosis foci counted under 20x microscope): 0 points, none; 1 point (< 2 foci); 2 points (2 to 4 foci); 3 points (> 4 foci). Hepatocellular ballooning: 0 points, none; 1 point, rare; 2 points, common.
The NAS score is obtained by adding the above three scores of hepatocyte steatosis, hepatocyte ballooning, and intralobular inflammation.

S2. Detailed methods for detection of targeted BAs metabolic profiling in feces.

S2.1. Metabolites Extraction
A 25 mg aliquot of each individual sample was precisely weighed and transferred to an Eppendorf tube. After the addition of 1000 μL of extract solution (precooled at -40 °C, acetonitrile-methanol-water, 2:2:1), the samples were vortexed for 30 s, homogenized at 35 Hz for 4 min, and sonicated for 5 min in ice-water bath. The homogenate and sonicate circle were repeated for three times, followed by incubation at -40 °C for 1 h and centrifugation at 12000 rpm (RCF=13800(×g), R= 8.6cm) and 4 °C for 15 min. The resulting supernatants were transferred to LC-MS vials for UHPLC-MS/MS analysis.
S2.2. Standard Solution Preparation
Stock solutions were individually prepared by dissolving or diluting each standard substance to give a final concentration of 1 mg/mL. An aliquot of each of the stock solutions was transferred to a flask to form a mixed working standard solution. A series of calibration standard solutions were then prepared by stepwise dilution of this mixed standard solution (containing isotopically-labelled internal standard mixture in identical concentrations with the samples).
S2.3. UHPLC-PRM-MS Analysis
The UHPLC separation was carried out using an UHPLC System (Vanquish, Thermo Fisher Scientific), equipped with a Waters ACQUITY UPLC BEH C18 column (150 * 2.1 mm, 1.7 μm, Waters). The mobile phase A was 5 mmol/L ammonium acetate in water, and the mobile phase B was acetonitrile. The column temperature was set at 45 °C. The auto-sampler temperature was set at 4 °C and the injection volume was 1 μL.
A Orbitrap Exploris 120 mass spectrometer (Thermo Fisher Scientific) was applied for assay development. Typical ion source parameters were: spray voltage = +3500/-3200 V, sheath gas (N_2) flow rate = 40, aux gas (N_2) flow rate = 15, sweep gas (N_2) flow rate = 0, aux gas (N_2) temperature = 350 °C, capillary temperature = 320 °C.
The parallel reaction monitoring (PRM) parameters for each of the targeted analytes were optimized, by injecting the standard solutions of the individual analytes, into the API source of the mass spectrometer. Since most of the analytes did not show product ion acceptable for quantification, the precursor ion in high resolution was selected for quantification.
S2.4. Calibration Curves
Calibration solutions were subjected to UPLC-PRM-MS/MS analysis using the methods described above. Figure S1-Calibration summarizes the results for the calibration curves where y is the peak areas ratio for analyte/IS, and x is the concentration (nmol/L) for analyte. Least squares method was

used for the regression fitting. 1/x weighting was applied in the curve fitting since it provided highest accuracy and correlation coefficient (R^2). The level was excluded from the calibration if the accuracy of calibration was not within 80–120%.

S2.5. Limit of Detection (LOD) and Limit of Quantitation (LOQ)

The calibration standard solution was diluted stepwise, with a dilution factor of 2. These standard solutions were subjected to UHPLC-PRM-MS analysis. The signal-to-noise ratios (S/N) were used to determine the lower limits of detection (LLODs) and lower limits of quantitation (LLOQs). The LLODs and LLOQs were defined as the analyte concentrations that led to peaks with signal-to-noise ratios (S/N) of 3 and 10, respectively, according to the US FDA guideline for bioanalytical method validation.

S2.6. Precision and Accuracy

The precision of the quantitation was measured as the relative standard deviation (RSD), determined by injecting analytical replicates of a QC sample. The accuracy of quantitation was measured as the analytical recovery of the QC sample determined. The percent recovery was calculated as [(mean observed concentration) / (spiked concentration)] × 100%.

S3. Detailed methods for detection of non-targeted metabolic profile in serum.

S3.1. Metabolites Extraction

100 μL of sample was transferred to an EP tube. After the addition of 400 μL of extract solution (methanol: acetonitrile= 1: 1, containing isotopically-labelled internal standard mixture), the samples were vortexed for 30 s, sonicated for 10 min in ice-water bath, and incubated for 1 h at -40 ℃ to precipitate proteins. Then the sample was centrifuged at 12000 rpm(RCF=13800(×g),R= 8.6cm) for 15 min at 4 ℃. The resulting supernatant was transferred to a fresh glass vial for analysis. The quality control (QC) sample was prepared by mixing an equal aliquot of the supernatants from all of the samples.

S3.2. LC-MS/MS Analysis

LC-MS/MS analyses were performed using an UHPLC system (Vanquish, Thermo Fisher Scientific) with a UPLC BEH Amide column (2.1 mm × 100 mm, 1.7 μm) coupled to Q Exactive HFX mass spectrometer (Orbitrap MS, Thermo). The mobile phase consisted of 25 mmol/L ammonium acetate and 25 ammonia hydroxide in water （pH = 9.75） (A) and acetonitrile (B). The auto-sampler temperature was 4 ℃, and the injection volume was 2 μL.

The QE HFX mass spectrometer was used for its ability to acquire MS/MS spectra on information-dependent acquisition (IDA) mode in the control of the acquisition software (Xcalibur, Thermo). In this mode, the acquisition software continuously evaluates the full scan MS spectrum. The ESI source conditions were set as following: sheath gas flow rate as 30 Arb, Aux gas flow rate as 25 Arb, capillary temperature 350 ℃, full MS resolution as 120000, MS/MS resolution as 7500, collision energy as 10/30/60 in NCE mode, spray Voltage as 3.6 kV (positive) or -3.2 kV (negative), respectively.

S3.3. Data preprocessing and annotation

The raw data were converted to the mzXML format using ProteoWizard and processed with an in-house program, which was developed using R and based on XCMS, for peak detection, extraction, alignment, and integration. Then an in-house MS2 database (BiotreeDB) was applied in metabolite annotation. The cutoff for annotation was set at 0.3.

Supplementary Table S1: Analysis of main components of fruit polyphenol by mass spectrometry

No.	tR(min)	[M-H]-	ppmError	Molecular formula	MS/MS	Compound
1	8.52	353.0880	3.7890	C16H18O9	191(100), 179(50), 135(80)	3-O-caffeoylquinic acid
2	8.91	353.0881	3.8665	C16H18O9	191(100), 179(60), 135(70)	5-O-caffeoylquinic acid
3	9.28	353.0879	3.6072	C16H18O9	191(80), 179(30), 135(100)	4-O-caffeoylquinic acid
4	10.87	431.1554	1.6213	C19H28O11	269(40), 161(30)	Apigenin-O-glucoside
5	10.94	447.0941	4.3815	C21H20O11	284(100), 255(30)	Kaempferol-3-O-glucoside
6	12.11	447.0927	1.2416	C21H20O11	284(100), 255(30)	Kaempferol-7-O-glucoside
7	12.16	289.0717	3.7740	C15H14O6	245(60), 203(90), 109(100)	Epicatechin
8	12.20	593.1513	2.1423	C27H30O15	284(100), 255(30)	Kaempferol-O-rutinoside
9	14.84	289.0717	3.7740	C15H14O6	245(20), 203(30), 109(100)	Catechin
10	22.45	609.1464	2.3268	C27H30O16	300(50), 271(40), 255(30), 179(5), 151(10)	Rutin
11	22.94	463.0886	3.2837	C21H20O12	301(100), 271(60), 255(30) , 243(30), 179(10), 151(20)	Hyperoside
12	23.32	463.0888	3.6791	C21H20O12	300(100), 271(100), 255(50) , 227(20), 179(5), 151(20)	Isoquercetin
13	23.90	463.0890	4.20663	C21H20O12	300(100), 271(70), 255(30) , 243(20), 179(5), 151(20)	Quercetin-7-O-glucoside
14	24.89	433.0778	3.1014	C20H18O11	300(100), 271(50), 255(30) 151(20)	Quercetin-3-O-arabinoside
15	26.28	433.0781	3.73560	C20H18O11	301(100), 271(70), 255(30) , 243(20), 179(5), 151(20)	Quercetin-7-O-arabinoside
16	26.81	447.0930	1.8560	C21H20O11	300(80), 271(70), 255(100)	Quercetin-O-rhamnoside
17	28.44	593.1513	2.1423	C27H30O15	447(5), 285(100), 229(30)	Kaempferol-7-O-rutin
18	33.15	301.0352	3.3193	C15H10O7	179(40), 151(80)	Quercetin

Supplementary Table S2: The primer sequences related to cholesterol metabolism and bile acids metabolism

Gene	Forward sequence (5’→3’)	Reverse sequence (5’→3’)
HMGCR	TGT TCA CCG GCA ACA ACA AGA	CCG CGT TAT CGT CAG GAT GA
LXRα	GCG TCC ATT CAG AGC AAG TGT	TCA CTC GTG GAC ATC CCA GAT
ABCA1	GCT TGT TGG CCT CAG TTA AGG	GTA GCT CAG GCG TAC AGA GAT
ABCG5	CGC GAG ACG TTG CGA TAC A	CTG CCA ATC ATT TGG TCC GC
ABCG8	CTG TGG AAT GGG ACT GTA CTT C	GTT GGA CTG ACC ACT GTA GGT
NPC1L1	TGT CCC CGC CTA TAC AAT GG	CCT TGG TGA TAG ACA GGC TAC TG
ACAT2	GAC TTG GTG CAA TGG ACT CG	GGT CTT GCT TGT AGA ATC TGG
CYP7A1	TTC AAG ACC GCA CAT AAA GCC	GAG ATG CCC AGA GGA TCA CG
CYP27A1	AGG AAG TGA CCC AGT TTG TGT T	GGT GTT GGA CCC TGG AGT TT
FXR	GCT TGA TGT GCT ACA AAA GCT G	CGT GGT GAT GGT TGA ATG TCC
SHP	CAG GTC GTC CGA CTA TTC TGT	AGG CTA CTG TCT TGG CTA GGA
BSEP	CTG CCA AGG ATG CTA ATG CA	CGA TGG CTA CCC TTT GCT TCT
NTCP	CTT GCG CCA TAG GGA TCT TC	TGC CTG CCT TGA GGA CGT A
ASBT	GTC TGT CCC CCA AAT GCA ACT	CAC CCC ATA GAA AAC ATC ACC A
FGF15	GAC CAA AAC GAA CGA AAT TTG TT	ACG TCC TTG ATG GCA ATC G
β-actin	CCT AGA AGC ATT TGC GGT GCA CGA TG	TCA TGA AGT GTG ACG TTG ACA TCC GT

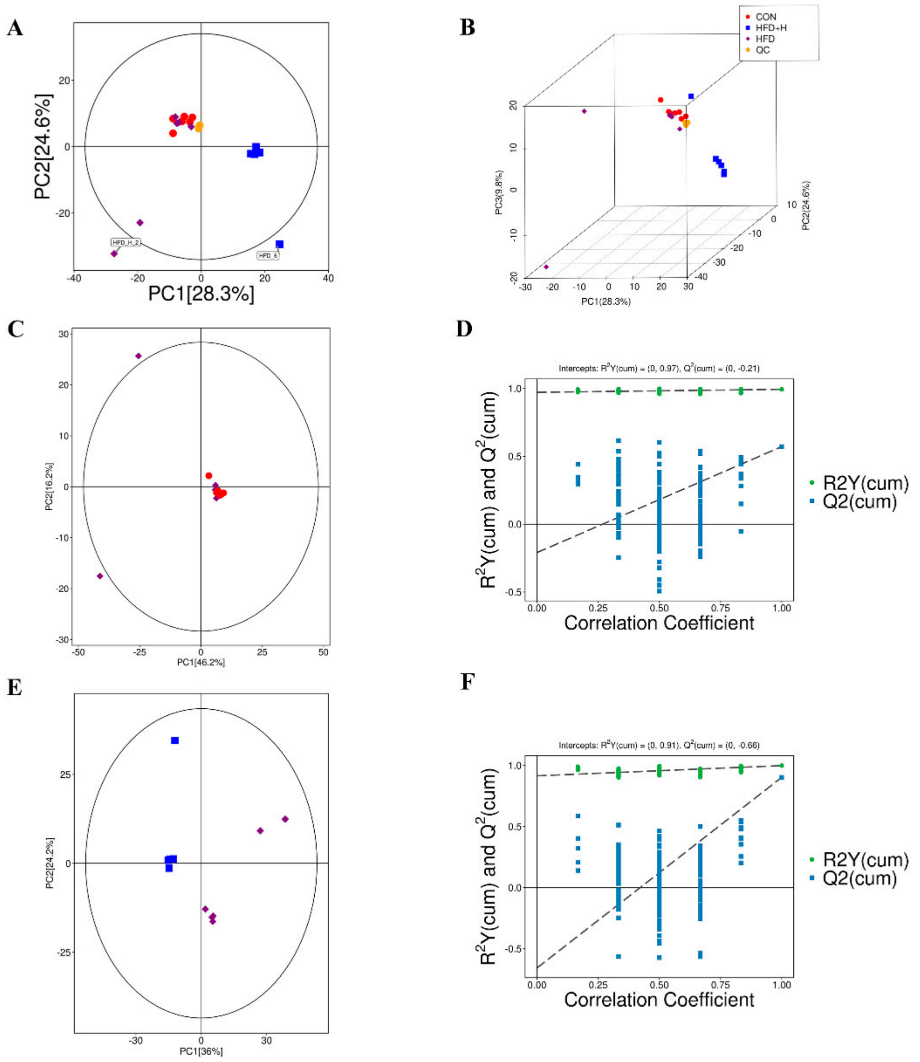

Supplementary Figure S1: Effect of PPE on serum metabolic profile. (A) PCA score plot and (B) PCA 3D score plot of serum metabolic profile of CK group, HFD group and HFD+H group. (C-D) PCA score plot and OPLS-DA score plot of serum metabolic profiles between CK group and HFD group. (E-F) PCA score plot and OPLS-DA score plot of serum metabolic profiles between HFD group and HFD+H group.
